# GFP's Mechanical Intermediate States

**DOI:** 10.1371/journal.pone.0046962

**Published:** 2012-10-31

**Authors:** John Saeger, Vesa P. Hytönen, Enrico Klotzsch, Viola Vogel

**Affiliations:** 1 Laboratory of Applied Mechanobiology, Department of Health Sciences and Technology, ETH Zürich, Zürich, Switzerland; 2 Institute of Biomedical Technology, University of Tampere and Tampere University Hospital, Tampere, Finland; Massachusetts Institute of Technology, United States of America

## Abstract

Green fluorescent protein (GFP) mutants have become the most widely used fluorescence markers in the life sciences, and although they are becoming increasingly popular as mechanical force or strain probes, there is little direct information on how their fluorescence changes when mechanically stretched. Here we derive high-resolution structural models of the mechanical intermediate states of stretched GFP using steered molecular dynamics (SMD) simulations. These structures were used to produce mutants of EGFP and EYFP that mimic GFP's different mechanical intermediates. A spectroscopic analysis revealed that a population of EGFP molecules with a missing N-terminal α-helix was significantly dimmed, while the fluorescence lifetime characteristic of the anionic chromophore state remained unaffected. This suggests a mechanism how N-terminal deletions can switch the protonation state of the chromophore, and how the fluorescence of GFP molecules in response to mechanical disturbance might be turned off.

## Introduction

Since the discovery that GFP can be used to monitor gene expression and protein localization in living organisms [1], GFP and its mutants have become the most widely used fluorescence markers to identify how protein expression levels and their spatial organization within cells and tissues are altered. When mapping spatial distributions of GFP-tagged protein within cells, the lack of GFP fluorescence is typically interpreted as an absence of the tagged protein in that region. Since a large fraction of proteins outside and within cells are not free to move but are physically connected to other proteins, mechanical forces generated by cells or acting on cells can stretch and unfold proteins [2], [3], [4], [5], [6], [7]. Furthermore, and in the context of research in the field of mechanobiology, fluorescence resonance energy transfer (FRET) constructs exploiting GFP derivatives as optical force or strain probes, are becoming increasingly popular [8], [9], [10]. If GFP constructs are used as mechanical sensors, it is prudent to consider how stretching GFP derivatives might impact their fluorescence.

Although there have been a number of AFM studies of GFP [11], [12], [13], [14], [15], and computational simulations to analyze how GFP might unfold under tensile forces, including a self-organized polymer models [14], [16], a coarse-grained elastic network model [17], and an Ising-like model [18], we present here the first atomic-level structural models of the forced unfolding trajectories of GFP as derived from SMD simulations. To further study the putative spectroscopic properties of the mechanical intermediates where terminal peptide sequences were removed, and in analogy to previous approaches [19], we then used mutants of EGFP and EYFP designed to mimic the structures of the mechanical intermediate states seen in SMD simulations.

Optical probes are urgently needed to decipher how mechanical forces direct tissue organization during development as well as in homeostasis and disease, which are among the big unsolved questions in biology and regenerative medicine [5], [20], [21], [22], [23], [24], [25], [26]. The information provided here, particularly the structures of the mechanical intermediates, and the events that are associated with passing each of the associated energy barriers may be useful in designing new classes of fluorescent, FRET and GFP derived probes.

## Results

Steered molecular dynamics simulations [27] were used here to derive structural information regarding unfolding trajectories. Using protocols and methods described previously [28], [29], [30], the known crystal structure of GFP was immersed in a box filled with explicit water molecules. After equilibration for two nanoseconds, a constant tensile force was applied to the termini.

To find significant intermediate states in the unfolding pathway of GFP, we subjected the molecule to constant tensile forces ranging from 20 pN to 300 pN, on a timescale of tens of nanoseconds depending on the simulation. Although, due to limitations of computational resources, SMD simulations typically employ higher forces and shorter time scales than what are seen *in vitro* or *in vivo*, SMD and other computational techniques have been successfully applied to identify intermediate structural states in the unfolding path of many proteins and the underlying structural transitions [19], [27], [28], [29], [30], [31], [32], [33].

**Figure 1:**
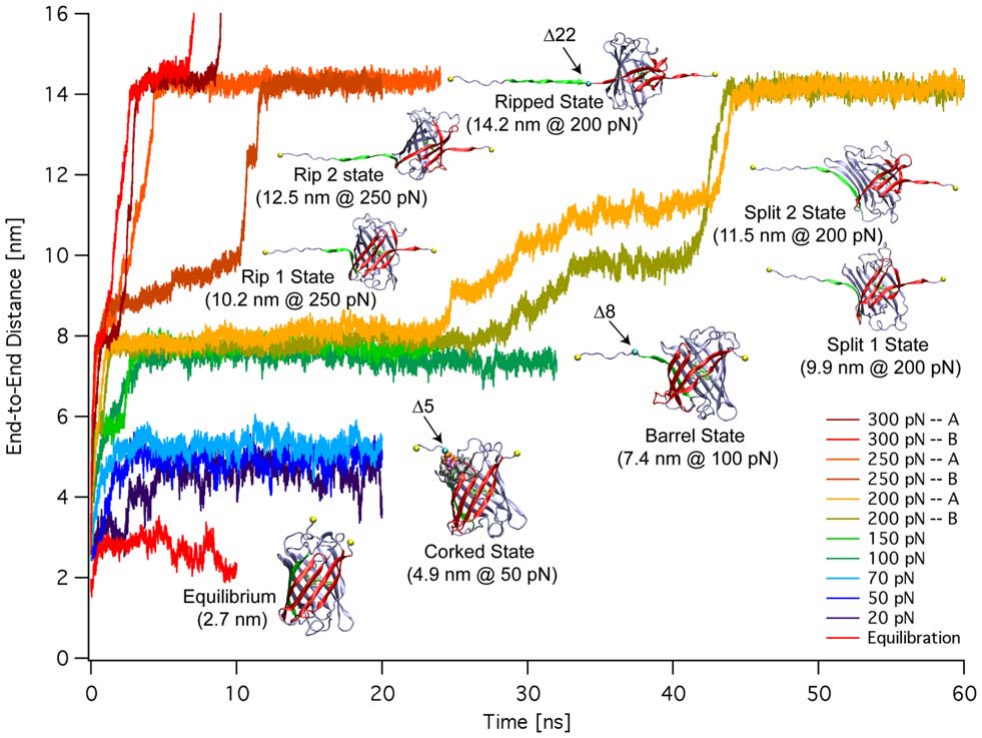
The main mechanical intermediates of GFP. The extension versus time graph for constant force pulls of GFP hydrated in a box of explicit water as derived by steered molecular dynamics simulations (SMD). Structures of the GFP molecule in significant mechanical intermediate states are shown. The major intermediates Corked, Barrel, and Ripped can be seen to have extended horizontal plateau regions compared to the others. The locations of the N-terminal deletions are indicated.

Several plateau regions exist in the extension-time plots (Figure 1) indicating the existence of multiple mechanical intermediate states separated by energy barriers that have to be passed before further unfolding can occur. Three major intermediate states, observed in twelve independent simulations are described below.

### Corked State

**Figure 2:**
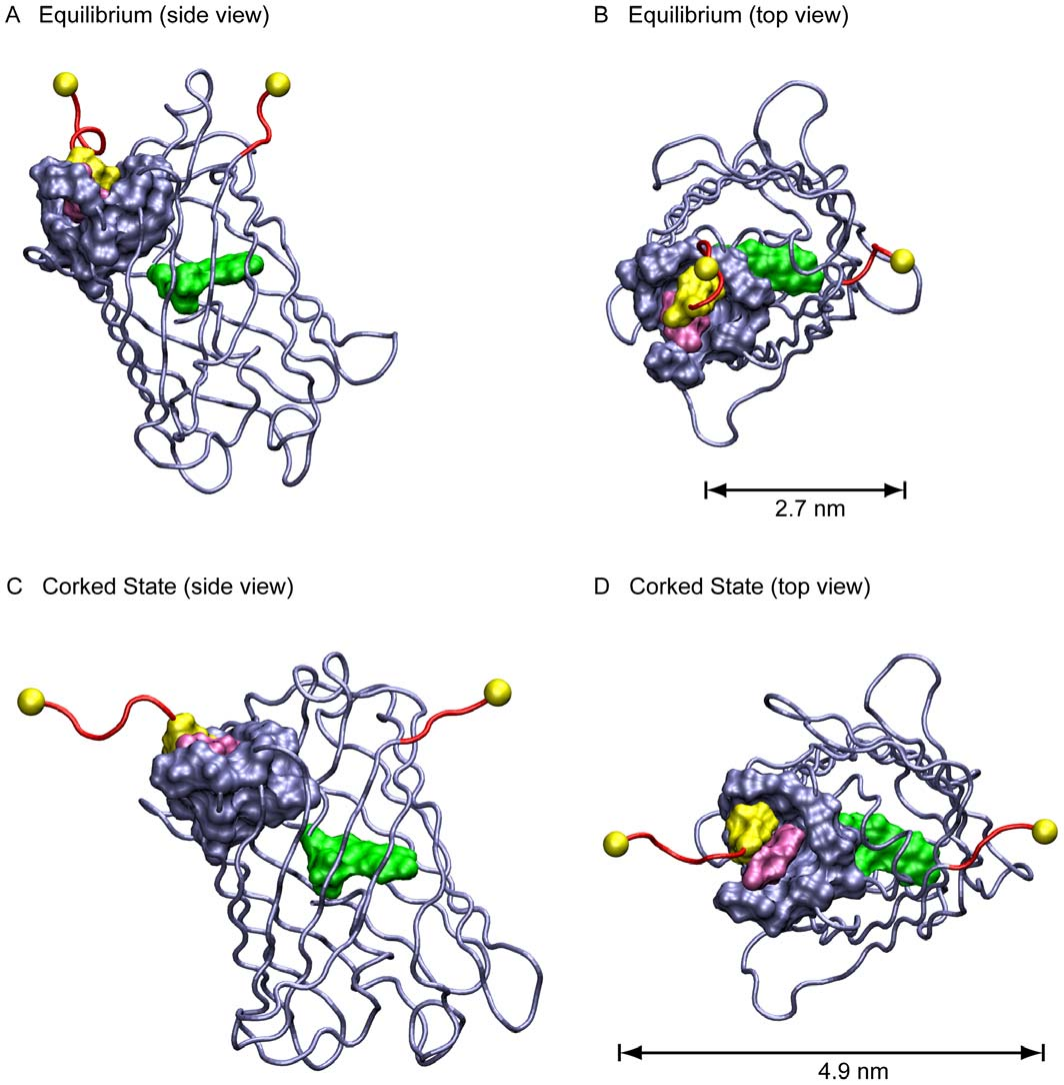
Transitioning into the Corked state. A, B. Two views of the equilibrium structure. C, D. Two views of the Corked state at 50 pN. The hydrophobic “cork” consisting of Leu7 and Phe8 has rotated in the hydrophobic pocket somewhat but remains bound. The mechanism is suggestive of a peptide “lever” with a hydrophobic “fulcrum.” GFP variants in this state likely remain significantly fluorescent (see text).

The first major intermediate state along the mechanical unfolding pathway of GFP is the Corked state (Figures 1, 2 and S1). Here, a portion of the short N-terminal α-helix consisting of residues 1 to 6 has pulled away from the barrel and has unraveled. The N-terminal part of the molecule that is still attached to the barrel and is stabilized with a hydrophobic “cork” consisting of Leu7 and Phe8 which is resting in a hydrophobic pocket on the surface of the protein barrel consisting of Pro89, Met88, Lys85, Cys70, Phe71, Ala37, Val12, Thr9, Phe114, Leu119 and Gly10. The backbone hydrogen bond between Gly10:O and Ala37:N, which does not exist in the crystal structure, was formed as the molecule was pulled into this state. It then broke again at approximately the same time as the “cork” was pulled out of the barrel. An examination of the structure in this state revealed that the hydrophobic “cork” consisting of residues Leu7 and Phe8 plays an additional role as a hydrophobic “fulcrum” around which the peptide “lever” consisting of the N-terminal residues 1–10 rotates. This facilitated the stabilization of the Gly10:O-Ala37:N hydrogen bond by the applied force, until this part of the structure unraveled.

Within the nanosecond timescale window of our simulations, one can pull the protein into the Corked state with a force as small as 20 pN, which was the smallest force explored in these simulations. The Corked state was also seen to remain stable at forces up to 70 pN for times approaching 20 ns, which was the maximum simulation time used for the pulls at these low forces, although at 70 pN the Corked state showed a partial extension of the C-terminus of the molecule where the hydrogen bonds between Ala227 and Tyr200 were broken as well as the Gly40:O-Val224:N hydrogen bond which at lower forces remained stable.

### Barrel State

**Figure 3:**
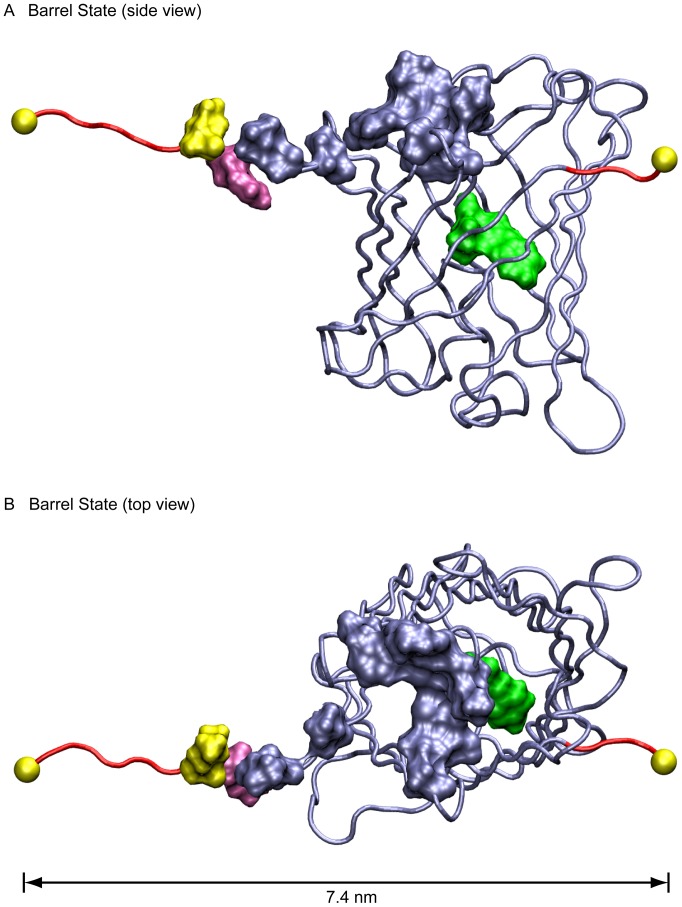
Transitioning into the Barrel state. A, B. Two views of the Barrel state at 100 pN. The “cork” consisting of Leu7 and Phe8 has been extracted from the hydrophobic pocket, and the hydrophobic pocket is significantly damaged. Nevertheless when pulled into this state, depending on the GFP variant, the molecule may retain significant fluorescence, or it may become almost completely dark (see text).

The Barrel state is entered when the hydrophobic cork consisting of residues Leu7 and Phe8 are pulled out of the hydrophobic pocket at the top of the protein barrel leaving the remainder of the barrel largely intact (Figures 1, 3 and S2). Here the N-terminal β-strand has continued to rip away from the barrel with residues 1–13 detached where the backbone hydrogen bonds between Val12 and Gly35 have broken as well as the Pro13:O-Leu119:N hydrogen bond. This leaves the N-terminus supported by backbone hydrogen bonds between Ile14 and Gly33 and the Leu15:O-Asn121:N hydrogen bond.

The two simulations that resulted in Barrel intermediate states (100 pN and 150 pN) displayed two different patterns of hydrogen bonds at the C-terminus and how they were broken. In the pull at 150 pN, the C-terminus retained its native conformation with hydrogen bonds between Tyr200 and Ala227 stabilizing the structure. In the pull at 100 pN, these hydrogen bonds rearranged such that the Tyr200:N-Ala227:O bond was broken, the Tyr200:O-Ala227:N bond was significantly weakened, with both being largely replaced by bonds between Tyr200 and Gly228 which are Tyr200:N-Gly228:O and Tyr200:O-Gly228:N.

### Ripped States

Beyond the Barrel state, there is a bifurcation of forced unfolding pathways. At forces of 250 pN and above, the N-terminal β1 strand rips cleanly away from the protein barrel, breaking bonds with neighboring strands β2 and β6 in an approximately symmetrical fashion (Figure 1). At 200 pN, a sizable gap opens between strands β1 and β2 prior to the detachment of strand β1 from strand β6. Although the pathways are different, eventually the bonds between strands β1 and β6 break in the pulls at 200 pN, and the pathways converge again with strand β1 completely detached from the barrel of the protein.

After complete detachment of the β1 strand along one or another pathway, the resulting structures were quite similar; differing mainly in the number of new hydrogen bonds formed between the β2 and β6 strands with some slight variation in the hydrogen bond pattern between the terminal strands and the barrel itself. We termed this ensemble of structures with high similarity the Ripped states. In most cases at 200 pN and 250 pN, where the Ripped states remained stable for multiple nanoseconds. After the extraction of the β1 strand, the β2 and β6 strands came together forming as many as six new hydrogen bonds. This resealing behavior, where a new ten-stranded β-barrel was formed while the protein was mechanically stressed, with mutually supporting β-strands aligned in a shearing configuration rather than an unzipping configuration [34], [35], [36], may play a key role in the mechanical strengthening of these intermediate states [37].

**Figure 4:**
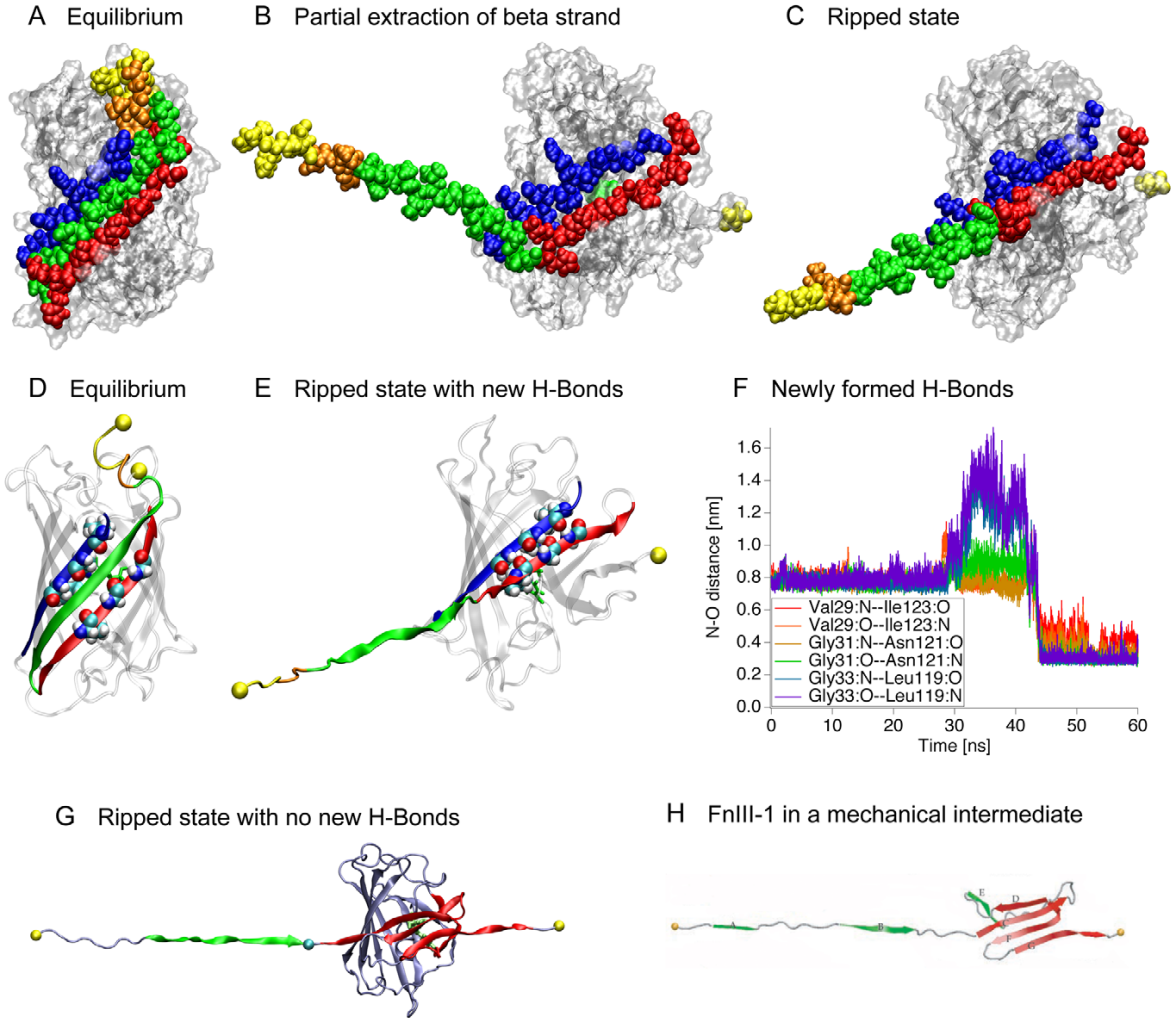
Transitioning into the Ripped states. A. The initial structure in a space-filling representation prior to pulling, with the β1 (green) strand nestled between the darker colored β6 (blue) and β2 (red) strands. The N-terminal α-helix is colored to illustrate the N-terminal deletions (yellow (Δ5) and orange (Δ8)). B. A partially unpeeled β1 strand leaving behind a gap between strands β6 and β2 through which the chromophore (green) can be seen. C. A completely extracted β1 strand with the β6 and β2 strands resealed. D. The initial configuration in the ribbon representation with the β1 strand colored in green. The amino acid residues that form new contacts are shown in the space filling representation. E. The resealed barrel. F. The distances between the atoms involved in hydrogen bond formation from the pull at 200 pN that best demonstrated resealing behavior. Contacts between Gly31-Asn121 as well as Gly33-Leu119 are more stable than the contacts between Val29-Ile123. G. The one case where the barrel did not reseal. However, there is a remaining stable “mechanical core” shown in red that bears a structural resemblance to an intermediate state of the first type III module from human fibronectin [38]. H. The first human fibronectin type III module (FnIII-1) in the intermediate state [38].

In one simulation, although the β2 and β6 strands came together, no new hydrogen bonds were formed during the observation window (Figure 4G). Nevertheless a structure is formed that bears a resemblance to a fibronectin type III module in a mechanical intermediate state (Figure 4H) [38]. In the Ripped states, N-terminal residues 1–27, and C-terminal residues 222–230 have detached from the barrel.

Seeing that the force-induced unfolding pathway of GFP is dominated by the removal of the N-terminal α-helix followed by the separation of the N-terminal β-strand from the main barrel of the protein, using a method similar to the one described previously [19], we modeled these force-induced mechanical intermediate states with genetically engineered N-terminal deletion mutants of EGFP and EYFP. This allowed us to characterize the putative spectroscopic properties of the mechanical intermediates by measuring populations of the mutants under equilibrium conditions. The Corked, Barrel, and Ripped states were modeled by deleting the first 5 (Δ5), 8 (Δ8), and 22 residues (Δ22) from the native structure respectively. Although these deletions are slightly shorter than the number of detached residues seen in the simulations, they were designed to mimic the dominant features of what were identified here as key mechanical intermediate states (Figure 1): the removal of the N-terminal α-helix with the hydrophobic cork intact (Corked) involving residues 1–6, the removal of the hydrophobic cork (Barrel) involving residues 7–13, and the detachment of the N-terminal β-strand (Ripped) involving residues 14–27. Since previous studies of N-terminal deletions of GFP [39], [40] were performed with unpurified proteins, cellular autofluorescence may have masked the small absorption and fluorescence signals. In our experiments, spectroscopy was performed on purified proteins.

**Figure 5:**
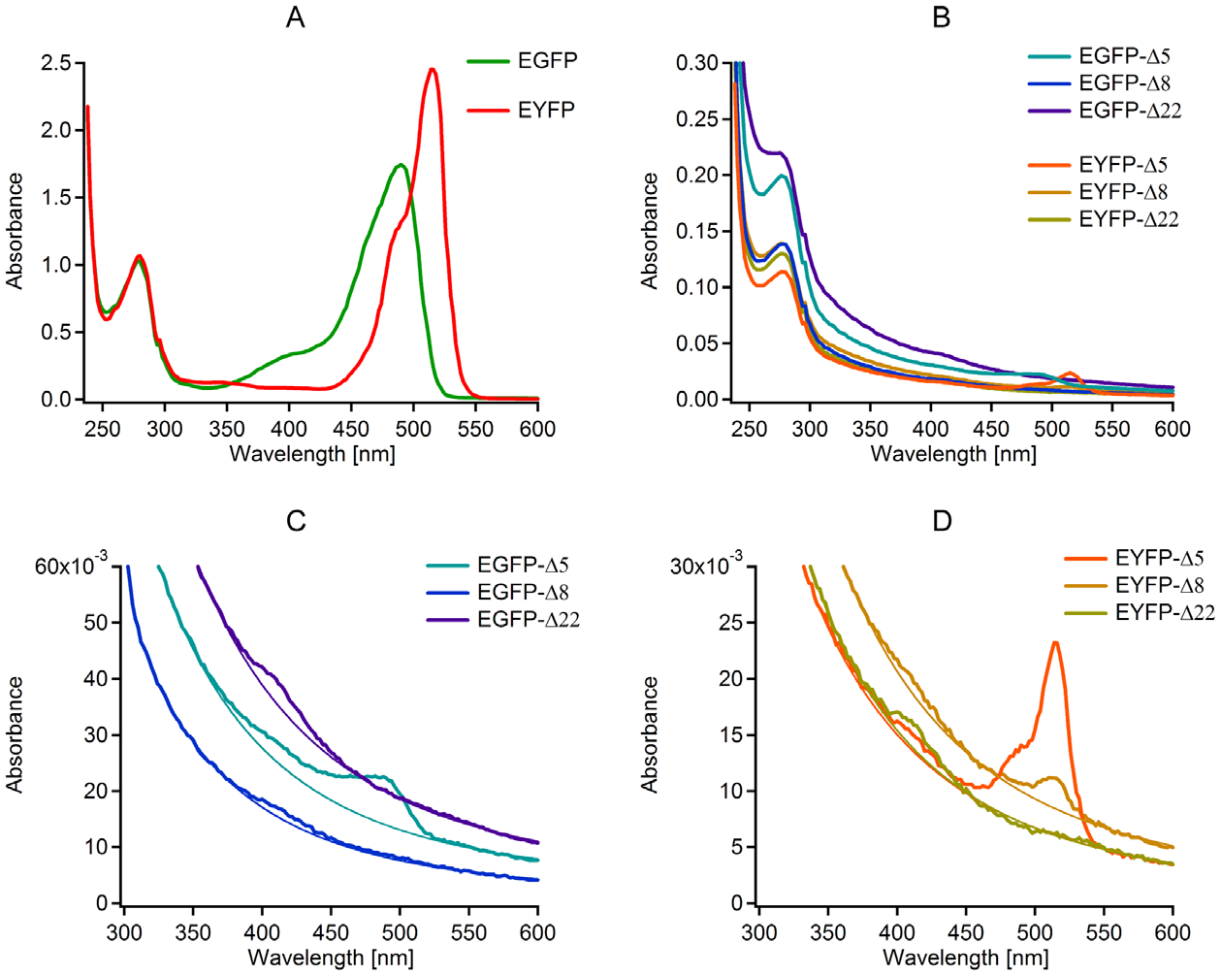
Absorption spectroscopy to probe for chromophore formation and relative brightness of the GFP mutants. A. Absorption spectra of the non-mutated proteins. B. Absorption spectra of the mutants. C, D. Absorption spectra of the mutants (zoomed). In addition to the absorption peak in the visible band, a weaker second peak is seen in the vicinity of 390 nm for all mutants, which corresponds to the absorption band of a neutral chromophore. Note the UV absorption peak in the Δ22 mutants. This indicates that a complete 11-stranded β-barrel is not needed to form a chromophore. The shapes of the absorption spectra displayed by the EGFP and EYFP Δ5 mutants are similar to the absorption spectra of the wild type molecules. This indicates that the Δ5 mutants are largely anionic similar to the wild type molecules and are therefore bright. The EGFP Δ8 mutant displayed no significant absorption peak in the visible band and is therefore very dim. The EYFP Δ8 mutant in addition to an absorption peak in the UV displayed a significant absorption peak in the visible band and is therefore only slightly dim. The Δ22 mutants displayed no significant absorption peak in the visible band and are therefore dark. The baseline function was fit to the background as described in the methods.

Since absorption spectroscopy can be used to detect the existence of a chromophore even in cases where it is non-fluorescent, we used it to probe chromophore formation in the mutants, and how their absorption spectra are altered when N-terminal residues are removed from the barrel (Figure 5). From the absorption spectra we derived estimates of the brightness of the deletion mutants that folded well enough to form a chromophore.

The existence of two absorption peaks, one in the near UV region, and another in the visible region, is a consequence of the fact that the EGFP and EYFP chromophores can both exist in two distinct states as regulated by shifting the state of their protonation. The neutral protonated chromophore has an absorption peak in the UV region, and the anionic deprotonated chromophore has an absorption peak in the visible region [41], [42]. The wild type chromophores of EGFP and EYFP exist largely in the anionic form with the major absorption peak being in the visible region. This anionic state gives rise to bright fluorescence emission, whereas the neutral state is almost completely non-fluorescent when excited in the visible region.

For the deletion mutants, the UV absorption peak became stronger relative to the visible peak as the length of the deletion increased, with the absorption peak of the anionic state completely missing in both of the Δ22 mutants (Figure 5). The existence of absorption in either the UV or visible bands indicates chromophore formation in at least a fraction of all of the mutants, which is consistent with results obtained with other partial GFP deletion mutants [43]. The Δ5 mutants had spectra with strong anionic absorption peaks similar to the wild type molecules. Assuming that the extinction coefficients and the quantum efficiency of the deletion mutants do not change relative to the wild-type molecules (see below), this indicates that these mutants are bright. The Δ8 mutants of EGFP and EYFP behave differently. The EGFP Δ8 mutant did not display a strong anionic absorption peak indicating that it is quite dim. On the other hand, the Δ8 mutant of EYFP showed a significant anionic absorption peak relative to the UV absorption peak indicating that it retains significant brightness.

Since subtle changes in the structure of the β-barrel might be a sign that other spectroscopic properties of the molecule such as the extinction coefficient or quantum efficiency have changed, we performed emission spectroscopy to determine if such changes in the structure of the β-barrel might be detectable in the fluorescent population of molecules.

**Figure 6:**
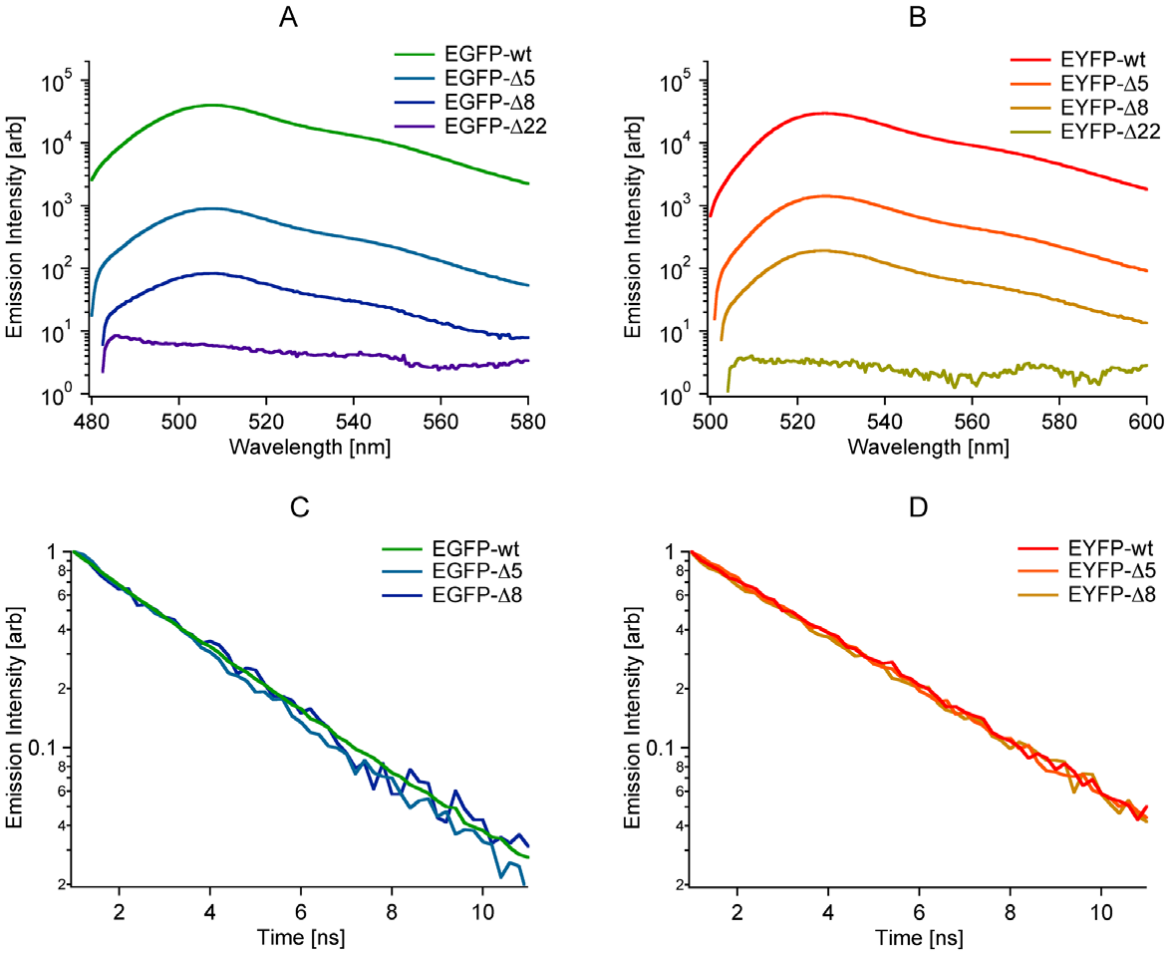
Emission spectroscopy to probe for changes in the β-barrels of the GFP mutants. A, B. Emission spectra of EGFP and EYFP based molecules excited at 460 nm and 480 nm respectively. Intensities were normalized to the absorbance of the molecules at 280 nm. The Δ22 mutants are seen to be completely dark when excited in the visible band. No significant changes in peak wavelength that would indicate changes to the structure of the β-barrel were observed [44]. C, D. Fluorescence lifetime decay curves of EGFP and EYFP based molecules. Intensities were normalized at a time approximately 1 ns after the laser pulse. No significant changes in fluorescence lifetime that would indicate changes to the structure of the β-barrel were observed [45].

First we performed emission spectroscopy with the molecules excited in the visible band (Figures 6A and 6B). Portions of all mutants were fluorescent except for the mutants corresponding to the Ripped state (Δ22), which were completely dark. All spectra appeared normal and no significant changes in the wavelength of the emission peak were observed. Changes in the wavelength of the emission peak have previously been linked to changes in the structure of the β-barrel [44]. To further examine the possibility that the structure of the β-barrel might be changing as the length of the N-terminal deletion was increased, we performed fluorescence lifetime measurements on the deletion mutants (Figures 6C and 6D). No significant changes in the lifetime of the excited states of the fraction of molecules that still emitted fluorescent light were observed. Thus we found no evidence that might indicate changes in the structure of the β-barrel of the progressively smaller fraction of fluorescent deletion mutants compared to the wild type molecules [45].

## Discussion

**Figure 7:**
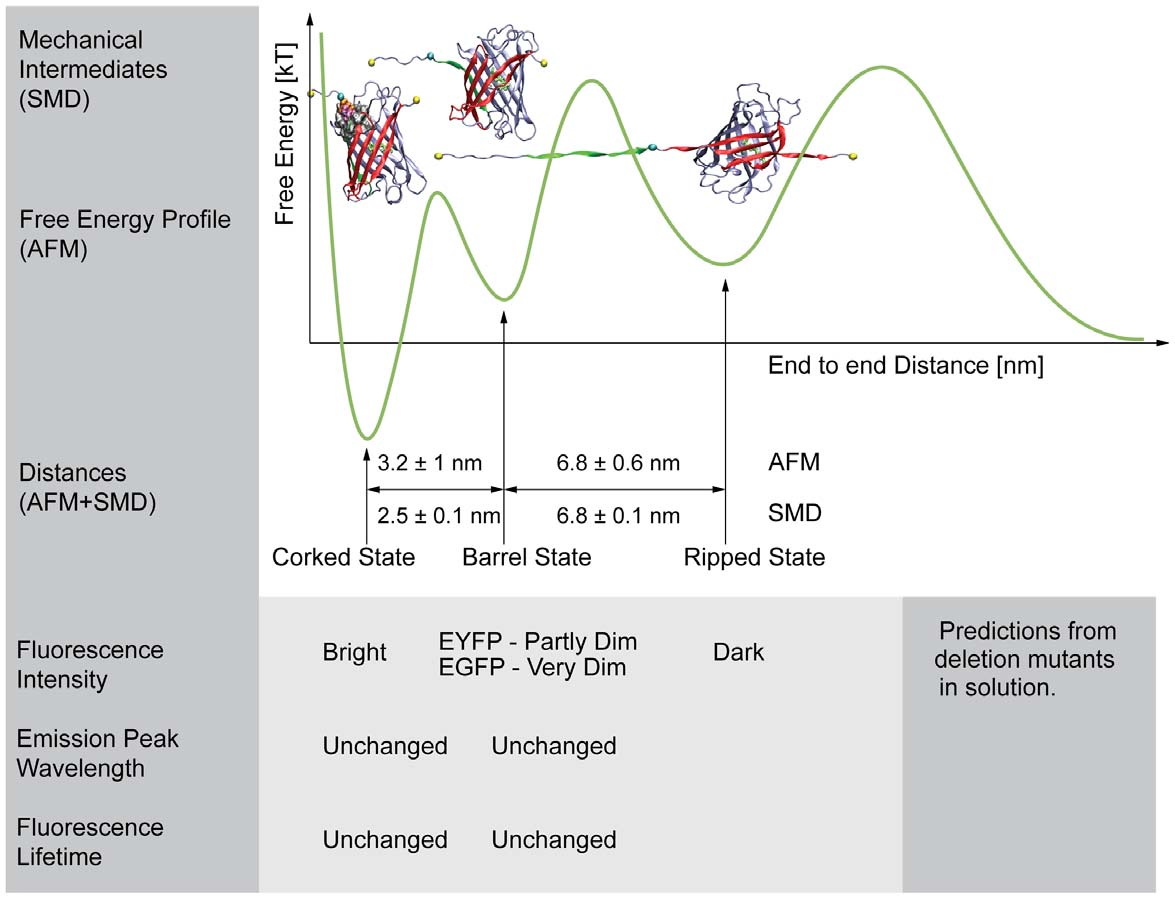
Correspondence of GFP's three major mechanical intermediate states between AFM and SMD [**13**]. The measurements of distance changes between mechanical intermediate states with SMD agreed with AFM [13] measurements within the uncertainties of the measurements. The pattern of decreasing brightness of the mutant EGFP and EYFP molecules which were designed to mimic the structure of the molecules in the significant mechanical intermediate states were consistent with the reduced thermodynamic stability of these states measured by AFM [13]. As we did not detect a significant energy barrier between the native state and the SMD corked state, we show the Corked state correlated with the bottom of the first energy well. The SMD Barrel state corresponds to the AFM [13] GFPΔα state where the N-terminal α-helix has been pulled away from GFP's β-barrel. The SMD Ripped state corresponds to the AFM [13] GFPΔαΔβ state where β1 strand has been pulled out of the β-barrel. The free energy diagram is adapted from Dietz and Rief [13]. A summary of predicted fluorescence properties of the mechanical intermediate states based on measurements of the deletion mutants is also shown.

Considering the many physiological processes that are mechanically regulated, it is essential to gain an understanding of fluorescence properties of GFP and its derivatives in settings where the protein might be mechanically stretched. Although there have been a number of AFM studies of GFP [11], [12], [13], [14], [15], we present here the first atomic resolution structural models of the forced unfolding trajectories of GFP derived using SMD (Figure 1). Several structural intermediate states exist early in the unfolding pathway of GFP and its derivatives. Importantly, the distances between the mechanical intermediate states derived from SMD (Figure 1), are in good agreement with those measured by AFM [13] (Figure 7) thus validating the computational approaches. The simulations, however, provide us with detailed insights into the high-resolution structures of the intermediate states and the structural changes that are associated with the transitions between these intermediates. The SMD-derived Barrel state corresponds to the AFM GFPΔα state where the N-terminal α-helix has been pulled away from the β-barrel (Figure 3). The SMD Ripped states correspond to the AFM GFPΔαΔβ state where the β1 strand has been pulled out of the β-barrel (Figure 4).

To gain an understanding how the spectroscopic properties might be altered by peeling off the N-terminal α-helix, using a method similar to the one described previously [19], we have genetically engineered truncated GFP variants to mimic these mechanical intermediate states. This allowed us to study the putative spectroscopic properties of truncated intermediate states under equilibrium conditions (Figures 5 and 6). We conducted absorption spectroscopy since it allows us to probe the fraction of molecules that have folded well enough to form a chromophore, even if their chromophore is non-fluorescent. Absorption spectroscopy on a population of molecules revealed that the EGFP and EYFP chromophores are increasingly protonated as we gradually truncated their N-termini (Figure 5). Protonation is known to turn EGFP and EYFP into a non-fluorescent state [41], [42] when excited in the visible absorption band, whereas the anionic states of these molecules are bright. Consequently we interpret the changes in protonation of the mutants to be associated with the observed changes in their brightness (Figure 5).

The Δ5 mutants corresponding to the SMD Corked state showed strong anionic absorption peaks similar to the wild type molecules (Figure 5). This indicates that these mutants are unprotonated and thus bright. The EGFP Δ8 showed absorption of the protonated state, but little absorption of the anionic species, indicating that it is protonated and thus dim. In contrast, the EYFP Δ8 showed significant absorption of the anionic species indicating that it still retains a more significant brightness. Hence, for this Δ8 truncation, a higher fraction of EGFP's than EYFP's are already protonated. In contrast, the Δ22 mutants of both EGFP and EYFP corresponding to the SMD Ripped state showed no absorption of the anionic species indicating that they are both dark.

To investigate the possibility that changes in the protonation of the chromophore might be explained by subtle changes in the structure of the β-barrel, emission spectroscopy was performed (Figure 6) to determine if such changes might be detectable in the fluorescent population of molecules. No significant changes in the wavelength of the emission peak were detected among the mutants. Additionally fluorescence lifetime measurements were performed. No significant changes in the lifetime of the excited states of the mutants were observed (Figure 6). Thus there was no evidence of changes to the structure of the β-barrel of the deletion mutants in the fluorescent population of the molecules [44], [45].

The possibility that mechanical forces can turn off the fluorescence of GFP and its variants needs to be considered when using them in the context of molecular environments that are exposed to tensile forces, since the loss of FRET, for example, would then not solely reflect changes of the relative distance or orientation of a FRET pair. The detailed knowledge of the mechanical pathway leading to loss of fluorescence presented here also suggests novel ways how to perhaps engineer a new generation of mechanically stabilized GFP probes. In case of fibronectin type III modules, it has been shown how point mutations can be exploited to tune their mechanical stability [30]. Our simulations now suggest the largest barrier to loss of fluorescence is the barrier stabilizing the GFP Corked state. This state is stabilized by the presence of hydrophobic residues Leu7 and Phe8 in a hydrophobic pocket on the surface of the protein. It is possible that mutations at these positions to residues that are slightly less hydrophobic, or a less perfect fit in the pocket would result in a protein whose fluorescence is more sensitive to mechanical disturbance.

Alternatively, FRET sensors made with GFP derivatives may profit from the use of fluorescent proteins that are less sensitive to mechanical disturbance. The finding that the Δ8 mutant of EYFP retained significant brightness where the Δ8 mutant of EGFP was very dim suggests that some GFP derivatives may be better suited for high mechanical stress or strain applications than others. The deletion mutant approach presented here may be of use to assess the stability and putative fluorescence of stretched GFP based molecules. Finally and in the light that GFP based FRET constructs are becoming increasingly popular [8], [9], [10] as mechanical strain probes, it should be noted that any force-induced unpeeling of β-barrels may furthermore contribute to distance changes between the FRET fluorophores (Figures 1 and 7).

## Materials and Methods

### Computational Methods

Computer simulations of GFP were based on an experimentally solved X-ray structure for the wild type GFP deposited in the Protein Data Bank (PDB) as 1GFL [46]. Molecular dynamics simulations were performed using protocols and methods similar to those previously described [27], [28], [29], [30], [31], [32], [38], [47], [48], [49], [50], [51], [52], [53], [54], [55]. Computations were carried out using the NAMD program [56] version 2.5 using the CHARMM22 force field [57] and the TIP3P explicit water model [58]. Simulation times ranged from 10 ns to 60 ns. The chromophore of the GFP molecule was modeled in its neutral form with CHARMM22 compatible force field parameters which were kindly provided by Nathalie Reuter [59]. The molecules were solvated in water boxes and aligned so that the line between the C-terminus and the N-terminus was parallel to the long axis of the box. There were at least 12 Å between the protein and the edge of the box in all dimensions prior to equilibration. Sodium and chloride ions were placed in the system at a concentration of 0.15 M, with enough additional sodium ions to balance the charge in the system. To accommodate the more extended intermediate states seen at higher forces, simulations at higher forces were performed in a longer box. Simulations at forces up to and including 150 pN were performed in a water box which had approximate dimensions of 9.6 nm×7.5 nm×7.5 nm and contained 17584 water molecules after equilibration. Simulations at forces higher than 150 pN were performed in a water box which had approximate dimensions of 15.5 nm×7.5 nm×7.5 nm and contained 29193 water molecules after equilibration.

Short-range nonbonded interactions were calculated to a distance of 12 Å with a switching distance of 10 Å. Electrostatic interactions were calculated with the Particle-Mesh-Ewald method [60] with approximately one grid point per Å^3^. A 1 fs time step was used with Berendsen pressure control at 1 atm and temperature control set to 310K with a coupling constant of 1 ps. After 2 ns of equilibration where free dynamics of the system was run without applied forces, the α-carbon of the carboxy-terminal residue and the α-carbon of the amino-terminal residue were subjected to constant forces equal in magnitude and opposite in direction.

### Design and Expression of DNA Constructs

The sequence for EYFP was obtained from pEYFP-N1 (Clontech, Mountain View, USA) and the EGFP sequence was obtained from pEGFP-N1 (Clontech). The region of interest was amplified by PCR. The 5′ primer contained EcoRI restriction enzyme cleavage site and the sequence encoding an N-terminal methionine followed by the sequence encoding N-terminus of EGFP/EYFP. Theoretical N-terminal sequences are as follows: WT, MASKGE; Δ5, MLFTGV; Δ8, MGVVPI; Δ22, MGHKFS. The 3′ primer had a BamHI cleavage site, which encodes amino acid residues GS. The C-terminal His_6_ purification tag was encoded by the pHIS plasmid sequence [61] followed by a stop codon.

The PCR-product was extracted from the agarose gel after electrophoretic separation. The restriction enzyme treated DNA insert and pHIS were then ligated with T4 ligase and transformed into *E. coli* DH5α. DNA sequencing was used to verify the expression constructs. As compared to wtGFP, EGFP has mutations F64L, S65T and R80Q and EYFP has mutations S65G, V68L, S72A, R80Q, and T203Y. The last GFP residue in the constructs used in this study is residue 230.

### Expression and Purification of Recombinant Proteins

EGFP, EYFP and N-terminal deletions thereof were prepared with C-terminal His-tags and were affinity purified. For protein isolation experiments, *E. coli* BL-21 (DE3) cells containing the expression construct of interest were cultured at 37°C overnight (shaking 150 rpm) in Lysogeny broth medium containing 50 µg/ml ampicillin and then diluted 1∶500 in fresh medium. Cultivation was continued until the OD_600_ reached 0.1 and temperature was then decreased to 26°C. To induce protein expression, 1 mM IPTG was added at OD_600_ 0.2–0.4 and shaking of the culture was continued overnight.

Cells were harvested after centrifugation (5000 g, 10 min), suspended in binding buffer (20 mM Tris-HCl, pH 7.9, 500 mM NaCl, 5 mM imidazole) and frozen. The cells were lysed with lysozyme at a concentration of 2 mg/ml and ultrasonicating the samples on ice. The lysate was clarified by centrifugation (15000 g, 15 min) followed by filtration with a 0.22 µm filter. The affinity purification was carried out using Ni-NTA sepharose packed in a 10 ml column. The column was equilibrated with binding buffer and lysate was loaded to the column, followed by a wash with binding buffer and washing buffer (20 mM Tris-HCl, pH 7.9, 500 mM NaCl, 20 mM imidazole). The elution was carried out with elution buffer (20 mM Tris-HCl, pH 7.9, 500 mM NaCl, 500 mM imidazole). Eluted proteins were dialyzed extensively against 10 mM Tris-HCl pH 8.0 containing 100 mM NaCl.

Wild type proteins were expressed efficiently and yielded 5–10 mg protein per liter of bacterial culture after affinity chromatographic purification. The protein yields from the deletion constructs were typically about 10–20% of that of wild type proteins.

### Absorption Spectroscopy

Absorbance measurements were performed with a Tecan infinite M200 UV/VIS spectrometer. Absorbance of the protein samples was measured in the 230–600 nm range. Chromophore formation was observed both at the normal absorption peaks for EGFP at 490 nm and EYFP at 514 nm as well as in the UV near 390 nm where absorbance of the neutral chromophore may be found. To facilitate the visualization of the absorption peaks of the deletion mutants, the analysis software Igor Pro (Wavemetrics, Inc., Lake Oswego, OR) was used to fit a power function of the form:
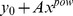
to the spectroscopic baseline using data at 340 nm, 344 nm, 550 nm, and 600 nm [62]. A plot of this function is shown in addition to the absorption data (Figures 5C and 5D).

### Emission Spectroscopy

Fluorescence emission spectroscopy was performed with a Perkin Elmer LS50B luminance spectrometer. Excitation was at 460 nm for EGFP based proteins and at 480 nm for EYFP based proteins.

Absorption and emission measurements were made at room temperature in a buffer consisting of 10 mM Tris-HCl at pH 8.0 containing 100 mM NaCl.

### Fluorescence Lifetime Spectroscopy

Fluorescence lifetime [63], [64], [65], [66], [67], [68] decay curves were measured on a home-built instrument similar to one described previously [69]. Briefly, a 470 nm solid-state pulsed laser (LDH-P-C-470, Picoquant, Germany) was used to illuminate the sample. The laser light was directed through a shortpass filter (3RD500SP; Omega Filters), reflected off a dichroic mirror (XF2010, 505DRLP; Omega Filters) and finally through a high numerical aperture microscope objective (NA = 1.4; Zeiss) into the sample. Emitted light passed through the dichroic mirror and was then limited to a 500–560 nm passband with a longpass filter (3RD500LP; Omega Filters) in series with a shortpass filter (3RD560SP; Omega Filters). The fluorescence was detected with an avalanche photodiode (APD). The signal from the APD was fed to a time-correlated single photon counting module (Becker & Hickl SPC-402), with which the fluorescence decay curves were recorded.

## Supporting Information

Figure S1 **Transitioning into the Corked state.** A, B. The Gly10:0-Ala37:N hydrogen shown in its open conformation at the beginning of the equilibration runs. C. The hydrophobic “cork” consisting of Leu7 and Phe8 in the hydrophobic pocket consisting of Thr9, Gly10, Val12, Ala37, Cys70, Phe71, Lys85, Met88, and Pro89 at the beginning of the simulations under force. D, E. The Gly10:O-Ala37:N hydrogen bond in its closed conformation after being closed by mechanical force. F. The hydrophobic “cork” after force-induced rotation. The mechanism is suggestive of a peptide “lever” with a hydrophobic “fulcrum.”
(TIF)

Figure S2 **Transitioning into the Barrel state.** A, C. Two views of the Barrel state at 100 pN. Compared to the native state, the Arg73:O-Ala226:N hydrogen bond is broken, and there is a rearrangement of hydrogen bonds involving Ala227, Tyr200, and Gly228 at the C-terminus of the protein. Gly228 has largely replaced Ala227 as the hydrogen bond partner with Tyr200, reinforcing the C-terminal strand. The Ala227:O-Tyr200:N bond is completely broken and Ala227:N-Tyr200:O is substantially weakened with Ala227:N sharing the Tyr227:O atom with Gly228:N. B, D. Two views of the Barrel state at 150 pN. In this case, compared to the native state, the C-terminus is less disturbed. The Arg73:O-Ala226:N hydrogen bond is still formed and the native pattern of hydrogen bonds between Ala227 and Tyr200 persist. In the native state, Gly228 has no hydrogen bond partners.
(TIF)

Movie S1 **Modeling GFP.** Excerpts of three simulations at different forces illustrating how the fluorescence of the mechanical intermediate states may depend on the variety of GFP based molecule.
(MP4)
