# Supplementary figures and images for: GFP's Mechanical Intermediate States
Source: PLoS One. 2012 Oct 31;7(10):e46962. doi: 10.1371/journal.pone.0046962 (PMC3485268; doi:10.1371/journal.pone.0046962)

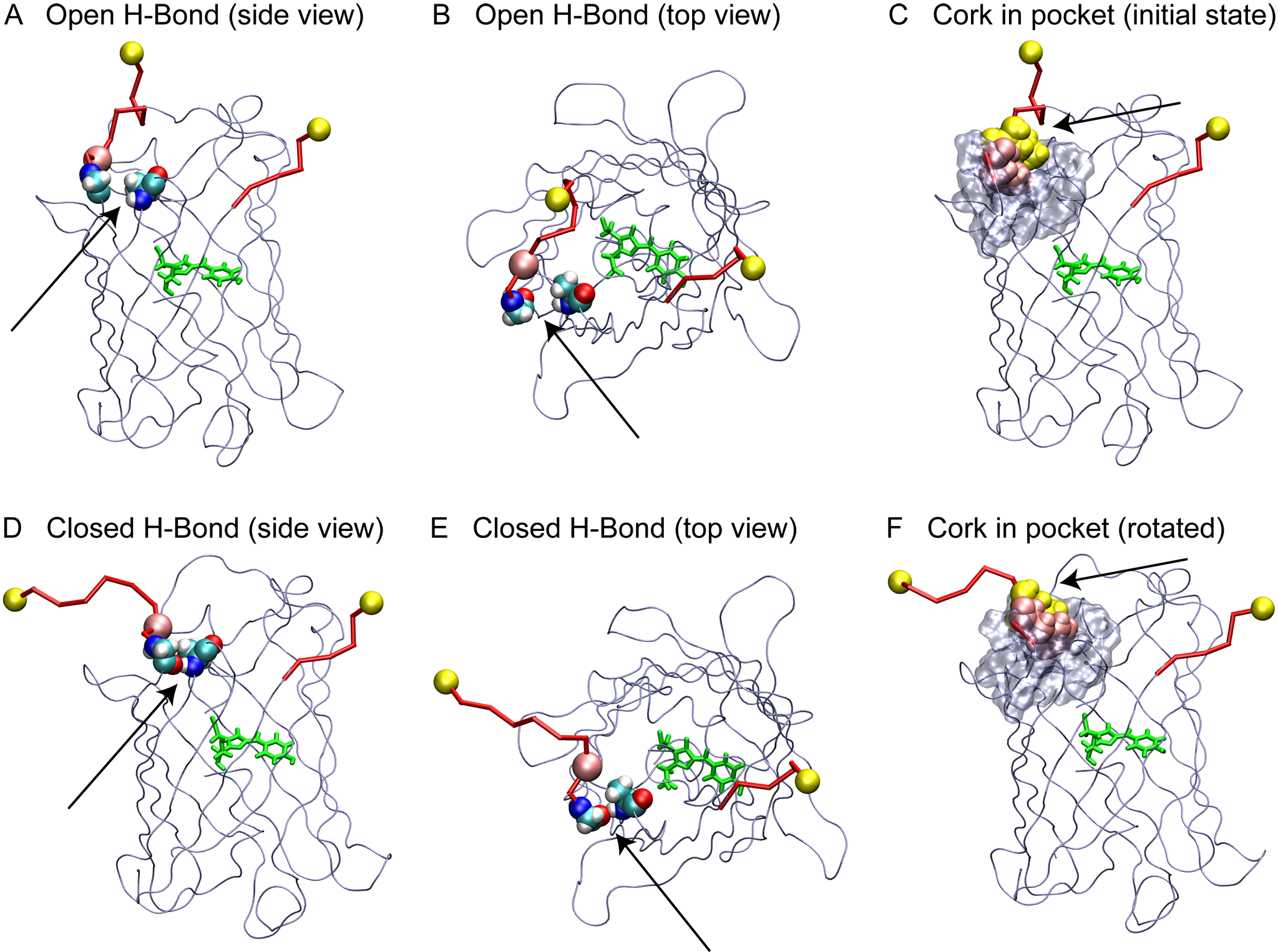

Supplement: Figure S1 — Transitioning into the Corked state. A, B. The Gly10:0-Ala37:N hydrogen shown in its open conformation at the beginning of the equilibration runs. C. The hydrophobic “cork” consisting of Leu7 and Phe8 in the hydrophobic pocket consisting of Thr9, Gly10, Val12, Ala37, Cys70, Phe71, Lys85, Met88, and Pro89 at the beginning of the simulations under force. D, E. The Gly10:O-Ala37:N hydrogen bond in its closed conformation after being closed by mechanical force. F. The hydrophobic “cork” after force-induced rotation. The mechanism is suggestive of a peptide “lever” with a hydrophobic “fulcrum.” (TIF) [file pone.0046962.s001.tif]

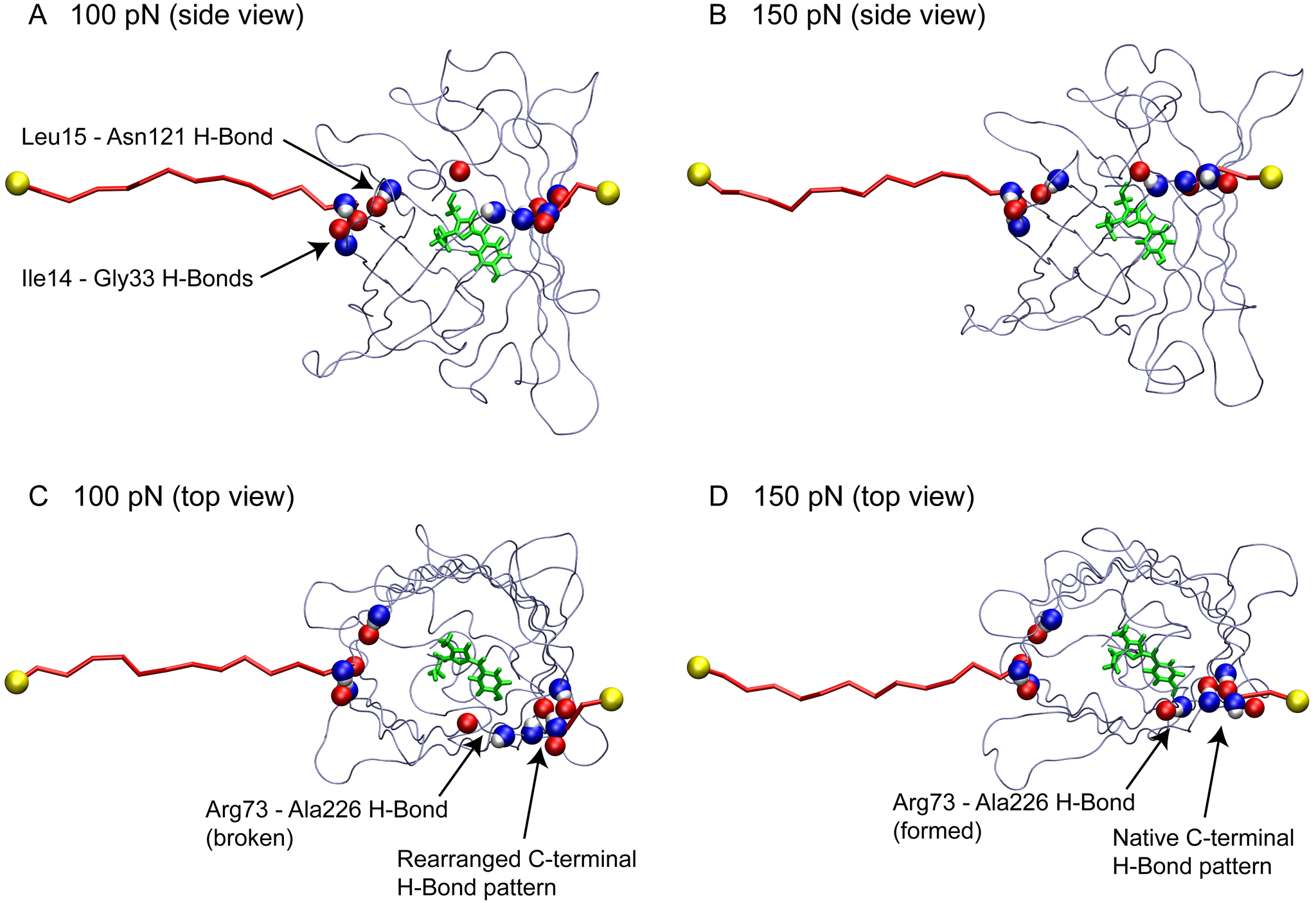

Supplement: Figure S2 — Transitioning into the Barrel state. A, C. Two views of the Barrel state at 100 pN. Compared to the native state, the Arg73:O-Ala226:N hydrogen bond is broken, and there is a rearrangement of hydrogen bonds involving Ala227, Tyr200, and Gly228 at the C-terminus of the protein. Gly228 has largely replaced Ala227 as the hydrogen bond partner with Tyr200, reinforcing the C-terminal strand. The Ala227:O-Tyr200:N bond is completely broken and Ala227:N-Tyr200:O is substantially weakened with Ala227:N sharing the Tyr227:O atom with Gly228:N. B, D. Two views of the Barrel state at 150 pN. In this case, compared to the native state, the C-terminus is less disturbed. The Arg73:O-Ala226:N hydrogen bond is still formed and the native pattern of hydrogen bonds between Ala227 and Tyr200 persist. In the native state, Gly228 has no hydrogen bond partners. (TIF) [file pone.0046962.s002.tif]
